# Sex‐based differences in track running distances of 100, 200, 400, 800, and 1500m in the 8 and under and 9–10‐year‐old age groups

**DOI:** 10.1002/ejsc.12075

**Published:** 2024-02-05

**Authors:** Gregory A. Brown, Brandon S. Shaw, Ina Shaw

**Affiliations:** ^1^ Physical Activity and Wellness Laboratory Department of Kinesiology and Sports Science University of Nebraska at Kearney Kearney Nebraska USA; ^2^ School of Sport Rehabilitation and Exercise Sciences University of Essex Colchester UK

**Keywords:** athletics, children, female, gender, male

## Abstract

There are contradictory claims regarding sex‐based differences in athletic performance before puberty, but there has been minimal evaluation of sex‐based differences in competitive running performance before puberty. The purpose of this project was to determine if there are prepubertal sex‐based differences in track running performance. Finalist times from the USA Track and Field National Youth Outdoor Championships and National Junior Olympic Championships during the years 2016–2023 for running distances of 100, 200, 400, 800, and 1500m in the 8 and under and 9–10‐year‐old age groups were analyzed for sex‐based differences. In the 8 and under age group, the males were, on average, faster (*p* = 0.000, Hedges' g = 0.632–0.834) than females by 4.0% in the distance of 100m, 4.7% in 200m, 5.3% in 400m, 6.7% in 800m, and 6.1% in 1500m. In the 9–10‐year‐old age group, the males were, on average, faster (*p* = 0.000, Hedges' g = 0.584–1.089) than females by 2.9% in the distance of 100m, 4.6% in 200m, 4.0% in 400m, 4.0% in 800m, and 5.9% in 1500m. In each distance and age group between 2016 and 2023, the individual fastest male was faster than the individual fastest female by 3.7 ± 2.3%. The present data indicate that, in elite competition, males in the 8 and under and 9–10‐year‐old age groups typically run faster than females of the same age by 2.9%–6.7% for running distances of 100, 200, 400, 800, and 1500m. These findings are of particular importance as government agencies and sports organizations consider policies regarding single sex sports competition for youth.

## Highlights

- In the 8 and under age group for running distances of 100, 200, 400, 800, and 1500m, the male finalists (i.e., the 8 fastest based on qualifying heats) were 5.4 ± 1.1% faster than the female finalists
- In the 9–10‐year‐old age group for running distances of 100, 200, 400, 800, and 1500m, the male finalists (i.e., the 8 fastest based on qualifying heats) were 4.3 ± 1.1% faster than the female finalists
- Over the 7 years evaluated, the fastest males were 3.7 ± 2.3% faster than the fastest females, with the fastest male performance being faster than the fastest female in every event

## 1 INTRODUCTION

In the United States of America, the passage of the federal civil rights law known as Title IX, which was part of the Education Amendments of 1972, resulted in exponential increases in the participation of females in sports. For example, Tanaka et al. (Tanaka et al., 2021) reports a 261.7% increase in the number of females participating in high school sports since the passage of Title IX, which contrasts to an 11.4% increase in the number of males participating in high school sports during the same time period. Many sports leagues for prepubertal children are not separated by sex since the focus is on developing basic sports skills rather than competition (Wells & Arthur‐Banning, 2008). However, the rationale and need for sex‐segregated sports in youth has become a controversial topic in the past few years.

A 2012 report from the United States Centers for Disease Control and Prevention indicated that there were no differences between 6 and 11‐year‐old males and females in performance on physical fitness tests (Ervin et al., 2013). Similarly, Mizuguchi et al. (2021) reported that there were no prepubertal sex‐based differences in competitive weightlifting performance. Senefeld et al. (2019) reported that the top 5 females under the age 10 swim faster than comparable males, while there was no difference in the performance of the 10th–50th place females and males. Some scholars have stated that there are no differences in athletic performance between males and females prior to the onset of puberty, and that it is only the increased testosterone secretion during puberty that causes males to outperform females in athletic competition (Handelsman, 2017; Handelsman et al., 2018; Safer, 2022). The recently released consensus statement from the American College of Sports Medicine on *The Biological Basis of Sex Differences in Athletic Performance* describes the prepubertal sex‐based differences in athletic performance as “minimal” (Hunter et al., 2023). Relevant to the scholarly conversation regarding prepubertal sex‐based differences in athletic performance, a recent legal filing claims that there is a medical and scientific consensus that sex‐based differences in sports performance do not arise until after male puberty has occurred (Roe v. UHSAA, 2022), and in another legal filing it is argued that is “purely speculative” to suggest that there are sex‐based differences in sports performance before puberty (A.M. v. Indianapolis Public Schools, 2022). It is also argued in popular media that sex‐segregated sports in children should be abolished because of the purported consensus that there are no sex‐based differences in athletic performance before puberty (Mertens, 2022).

In contrast to the aforementioned scholarly, legal, and popular media information claiming that there are no sex based differences in physical fitness and athletic performance before puberty, a number of other sources indicate that there are. For example, the Presidential Fitness Test was widely used in schools in the United States from the late 1950s until 2013, and the data indicate that 8–10‐years‐old males run 1.8%–5.9% faster than females of the same ages in the 30‐foot shuttle run and 10.4%–14.7% faster in the mile run (President’s Challenge Qualifying standards). There are also a number of studies that have found that, as young as 6 years old, males consistently and significantly outperform females of the same age on various tests of muscular strength, muscular power, aerobic fitness, and muscular endurance (Catley & Tomkinson, 2013; Fühner et al., 2021; Tambalis et al., 2016; Tomkinson et al., 2017, 2018; Vanhelst et al., 2020). Similarly, Papaiakovou et al. (2009) reported that sedentary males, aged 7–18‐years‐old, ran a 30‐m sprint faster than comparably aged sedentary females. Furthermore, the overall youth records for all‐time best performances (as of December 19, 2018) from USA Track & Field (USATF, 2018) and from the USA Track & Field National Junior Olympic Track and Field Championships (as of March 27, 2019) for the 8 and under and 9–10‐year‐old age groups (USATF, 2019) the Amateur Athletic Union (as of November 22, 2023) for the 8 and under, 9‐years‐old, and 10‐years‐olds age groups (AAU, 2023), and the School Sport Australia Track & Field Championships (as of December 2016) for age 10 (School Sport Australia, 2016) indicate that males outperform females of the same ages in all running, jumping, and throwing events. In addition, the 10 and under records for all‐time best performances from USA Swimming (as of November 22, 2023) indicate that males are faster than females in 11 out of 12 individual short course events and 9 out of 11 individual long course events (USA Swimming, 2023). Previous scholarly evaluations of the differences in athletic performance between juvenile males and females have primarily focused on ages 11–18 years (for examples, see Huebner & Perperoglou, 2019; Katić et al., 2013; Tonnessen, Svendsen, Olsen, Guttormsen, & Haugen, 2015), with only a single known evaluation of competitive running performance in children under age 10 (Handelsman, 2017).

In Handelsman's 2017 paper (Handelsman, 2017), the author states that prepubertal males run 3.0% faster than females with the difference increasing to 10.1% at 12.4 years, but the author subsequently states that sex‐based differences in athletic performance do not arise until 12–13 years of age. Part of the discrepancy in the interpretation within Handelsman's paper could be that the data are drawn from a source of performance times for ages 5–19 years old, with sufficient data for analysis of only ages 9–19 years old, and running distances of 50, 60, 100, 200, 300, 400, 500, 600, 800, 1000, 1500m, 1 mile, 2000m, 3000m, and 2 miles pooled together for analysis.

Given the disagreement about whether or not there are differences in athletic performance before puberty, an evaluation of competitive running in prepubescent children is warranted. Therefore, the purpose of this project was to determine if there are sex‐based differences in track running performance for distances of 100, 200, 400, 800, and 1500m in elite athletes under age 10 (who can reasonably be assumed to be prepubertal).

## 2 METHODS

Official finalist times for males and females for the USA Track and Field (USATF) National Youth Outdoor Championships and the USATF National Junior Olympic Championships during the years 2016–2023 for running distances of 100, 200, 400, 800, and 1500m in the 8 and under age group were downloaded in 1‐year brackets from the Athletic.net website between January 1 and August 12, 2023. The USATF National Youth Outdoor Championships and the USATF National Junior Olympic Championships were not held in 2020 due to the COVID 19 pandemic. In the 1500m distance there typically were no preliminary and finalist heats, so the 8 fastest times were used. The same procedures were repeated for the 9–10‐year‐old age group. With five events with eight finalists for two age groups and two sexes over seven years, there were potentially 2240 finishing times for data analysis. Due to there not being eight athletes with finishing times for each event each year, there were 2181 finishing times included in the data analysis (Table 1). The 8 and under and 9–10‐year‐old age groups represent the youngest age divisions used in USATF events, and these ages represent children who are almost certainly prepubertal. The USATF National Youth Outdoor Championships and USATF National Junior Olympic Championships were selected for comparison as these track meets should represent a sampling of elite youth runners from across the USA and the meets should be conducted using uniform policies regarding eligibility and timing procedures. Distances of 100, 200, 400, 800, and 1500m were used since these are the individual events for both the 8 and under and 9–10‐year‐old age groups. All procedures for this project accessed public information and did not require ethical review in accordance with the Code of Federal Regulations, 45 CFR 46.102, and the Declaration of Helsinki.

**TABLE 1:** Events and number of finishing times analyzed in the 8 and under and 9–10‐year‐old age groups from the USA Track and Field National Youth Outdoor Championships and the USA Track and Field National Junior Olympic Championships during the years 2016–2023 (These track meets were not held in 2020 due to the COVID‐19 pandemic).

Event	Age group	Males		Females	
		*n*	Explanation for why *n* ≠ 112 (if necessary)	*n*	Explanation for why *n* ≠ 112 (if necessary)
100m	8 and under	110	1 DNS in 2018 in YC 1 DNS in 2019 in YC	112	
	9–10‐year‐old	110	1 DNS in 2018 in YC 1 DNS in 2019 in YC	111	1 DNS in 2023 in YC
200m	8 and under	109	2 DNS in 2017 in YC 1 DNS in 2019 in YC	112	
	9–10‐year‐old	111	1 DNS in 2018 in YC	111	1 DNS in 2021 in YC
400m	8–and–under	105	Only 6 finalists in 2018 in YC 1 DQ in 2022 in YC 2 DNS in 2023 in YC 1 DNS in 2018 in JO 1 DQ in 2023 in JO	108	1 DQ in 2019 in YC 1 DQ in 2022 in YC 1 DQ in 2019 in JO Only 7 finishers in 2021 in JO
	9–10‐year‐old	106	1 DNS in 2018 in YC 1 DNS and 1 DQ in 2019 in YC 2 DQ in 2021 in JO 1 DQ in 2022 in JO	110	1 DQ in 2017 YC Only 7 finishers in 2017 in JO
800m	8 and under	111	Only 7 finishers in 2018 in YC	104	1 DNS and 1 DQ in 2017 in YC 1 DQ in 2018 in YC Only 7 finishers in 2019 in YC Only 7 finishers and 1 received DNS in 2023 in YC 1 DNS in 2018 in JO Only 7 finishers in 2021in JO
	9–10‐year‐old	110	1 DQ in 2016 in JO 1 DQ in 2023 in JO	108	1 DNS in 2017 in YC 1 DNS in 2018 in YC 1 DQ in 2018 in JO 1 DNS in 2022 in JO
1500m	8 and under	105	Only four finalists in 2018 in YC Only five finalists in 2019 in YC	104	Only four finalists in 2019 in YC Only 7 finalists in 2023 with 3 receiving times of DNS in YC
	9–10‐year‐old	112		112	

*Note*: The finish times were taken from the finalist heat or top 8 finishers for events not run in heats. YC = USA Track and Field National Youth Outdoor Championships. JO = USA Track and Field National Junior Olympic Championships. DNS = Did Not Start. DQ = Disqualified.

### 2.1 Calculations and statistics

Data are presented as means ± standard deviation. All times were converted from minutes:seconds to seconds where necessary. Data for males and females in each group were compared using a 2‐tailed 2‐sample unequal variance *t*‐test (SigmaStat 4.0, Systat Software). The effect size was calculated using Hedges' g since the sample sizes in each sex were not always the same. Percent difference between the male and female performances in each event were calculated using the equation described by Millard–Stafford (Millard‐Stafford et al., 2018)

$$\frac{Male\;Time-Female\;Time}{Male\;Time}\;X\;100$$

## 3 RESULTS

Pooling together, the present data for all ages and distances indicates that the average times for males were 4.8 ± 1.2% faster than the females, and the fastest males were 3.7 ± 2.3% faster than the fastest females. In all events, the average and fastest times for males were faster than females.

**FIGURE 1:**
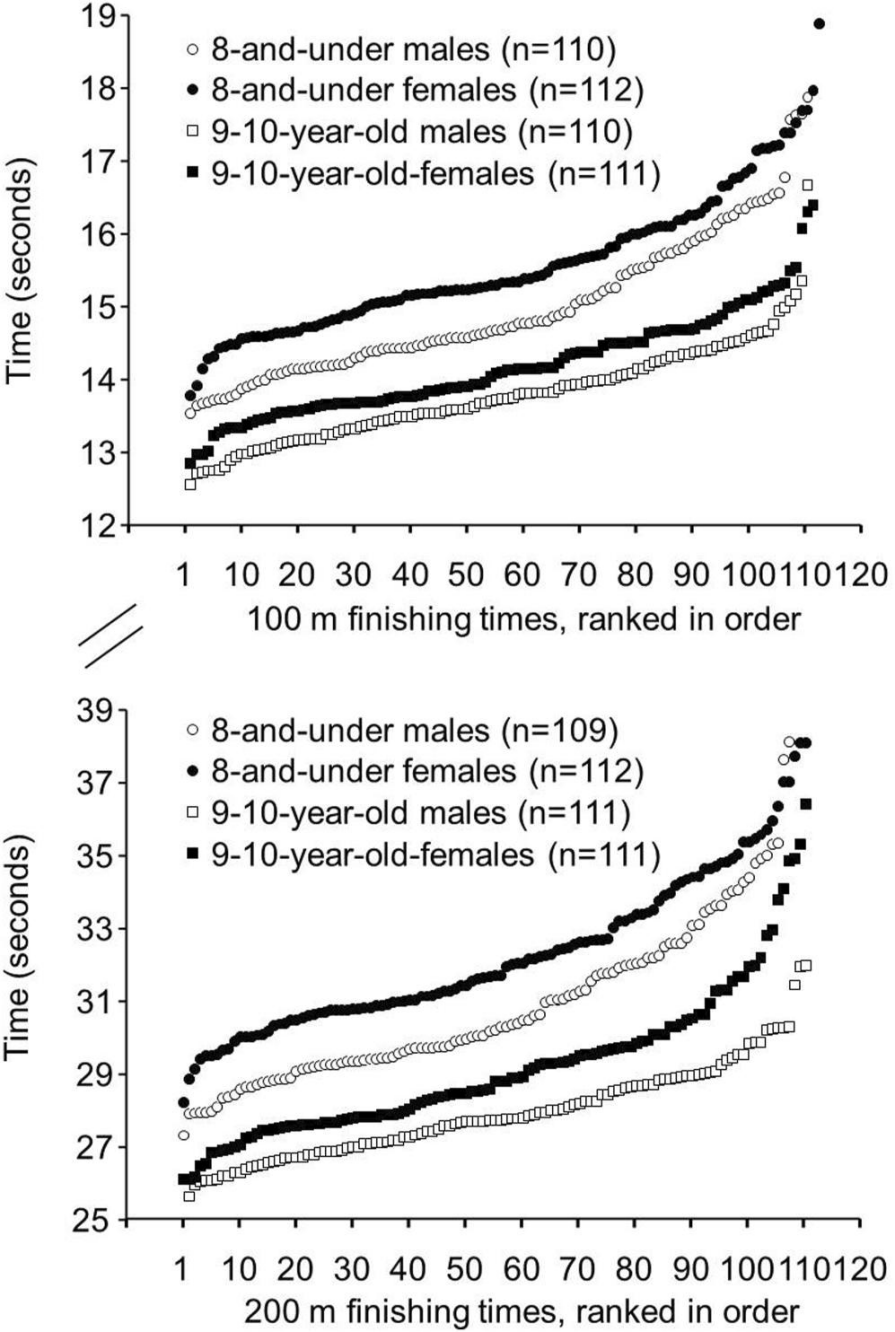
Upper Panel. Finishing times from fastest to slowest for the finalists in the 100m races in the 8 and under and 9–10‐year‐old age groups from the USA Track and Field National Youth Outdoor Championships and the USA Track and Field National Junior Olympic Championships during the years 2016–2023*. *p* = 0.000 for 8 and under males versus females, effect size (Hedges' g) = 0.632. *p* = 0.000 for 9–10‐year‐old males versus females, effect size (Hedges' g) = 0.584. Lower Panel: Finishing times from fastest to slowest for the finalists in the 200m races in the 8‐and‐under and 9‐10‐year‐old age groups from the USA Track and Field National Youth Outdoor Championships and the USA Track and Field National Junior Olympic Championships during the years 2016–2023*. *p* = 0.000 for 8 and under males versus females, effect size (Hedges' g) = 0.636. *p* = 0.000 for 9‐10‐year‐old males versus females, effect size (Hedges' g) = 0.738. *These track meets were not held in 2020 due to the COVID‐19 pandemic.

100 m: Finishing times in the 100‐m race for the 8 and under males (14.97 ± 0.97 s) were faster (*p* = 0.000; Hedges' g = 0.632) than for the 8 and under females (15.58 ± 0.96 s). The range of finishing times in the 100‐m race for the 8 and under males was 13.53–17.88 s (Figure 1, upper panel), while the range for the 8 and under females was 13.78–18.88 s. Eight males in the 8 and under age group had faster 100m finishing times than the fastest female in the 8 and under age group.

Finishing times in the 100‐m race for the 9–10‐year‐old males (13.78 ± 0.67 s) were faster (*p* = 0.000; Hedges' g = 0.584) than for the 9–10‐year‐old females (14.18 ± 0.70 s). The range of finishing times in the 100‐m race for the 9–10‐year‐old males was 12.55–16.77 s (Figure 1, upper panel), while the range for the 9–10‐year‐old females was 12.84–16.40 s. Seven males in the 9–10‐year‐old age group had faster 100m finishing times than the fastest female in the 9–10‐year‐old age group.

200 m: Finishing times in the 200m race for the 8 and under males (30.88 ± 2.31 s) were faster (*p* = 0.000; Hedges' g = 0.636) than for the 8 and under females (32.33 ± 2.25 s). The range of finishing times in the 200‐m race for the 8 and under males was 27.32–39.53 s (Figure 1, lower panel), while the range for the 8 and under females was 28.21–40.03 s. Seven males in the 8 and under age group had faster 200m finishing times than the fastest female in the 8 and under age group.

Finishing times in the 200‐m race for the 9–10‐year‐old males (27.90 ± 1.33 s) were faster (*p* = 0.000; Hedges' g = 0.738) than for the 9–10‐year‐old females (29.17 ± 2.04 s). The range of finishing times in the 200‐m race for the 9–10‐year‐old males was 24.68–31.98 s (Figure 1, lower panel), while the range for the 9–10‐year‐old females was 26.10–36.42 s. Six males in the 9–10‐year‐old age group had faster 200m finishing times than the fastest female in the 9–10‐year‐old age group.

**FIGURE 2:**
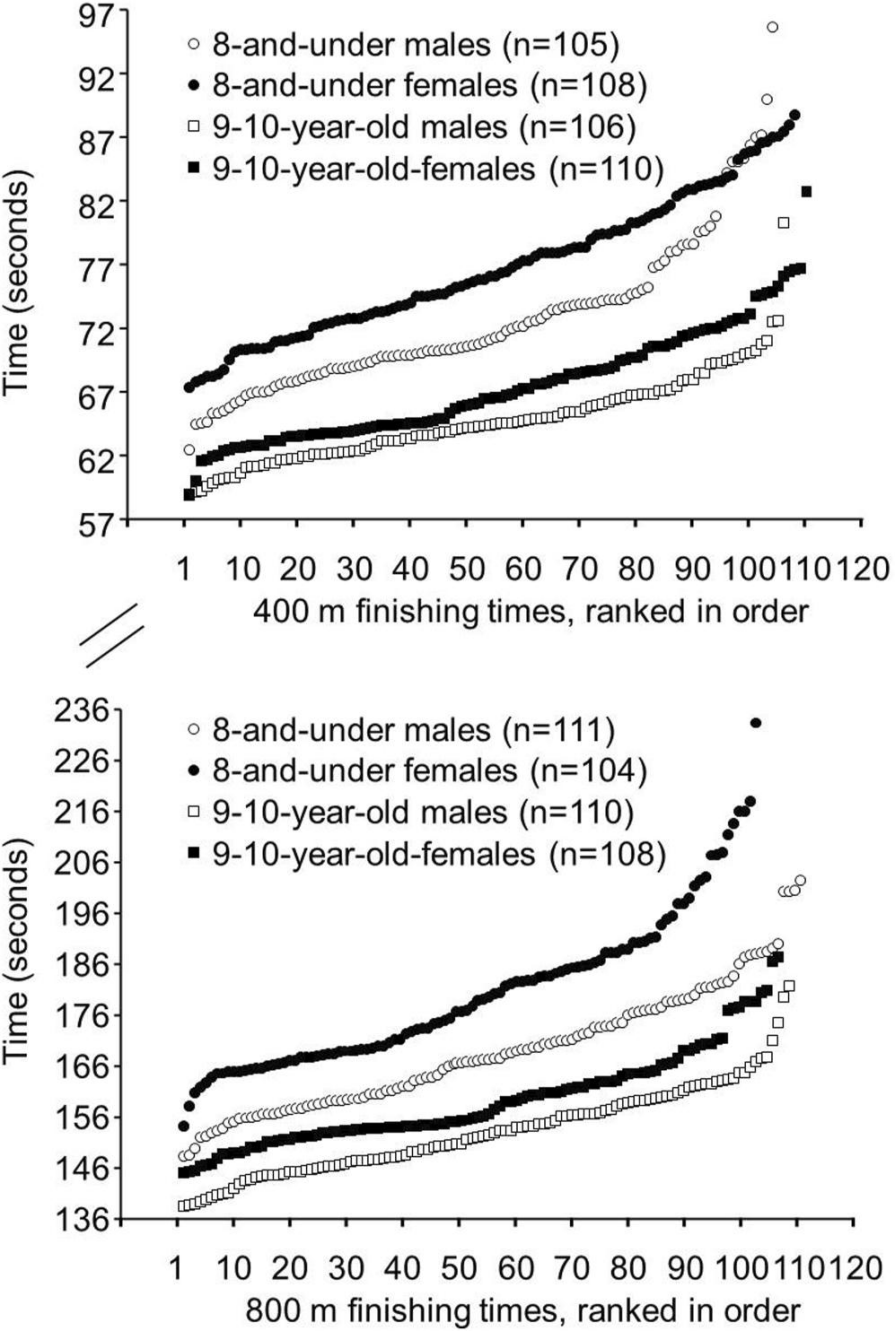
Upper Panel. Finishing times from fastest to slowest for the finalists in the 400‐m races in the 8 and under and 9–10‐year‐old age groups from the USA Track and Field National Youth Outdoor Championships and the USA Track and Field National Junior Olympic Championships during the years 2016–2023*. *p* = 0.000 for 8 and under males versus females, effect size (Hedges' g) = 0.640. *p* = 0.000 for 9–10‐year‐old males versus females, effect size (Hedges' g) = 0.664. Lower panel. Finishing times from fastest to slowest for the finalists in the 800m races in the 8‐and‐under and 9‐10‐year‐old age groups from the USA Track and Field National Youth Outdoor Championships and the USA Track and Field National Junior Olympic Championships during the years 2016–2023*. *p* = 0.000 for 8 and under males versus females, effect size (Hedges' g) = 0.834. *p* = 0.000 for 9‐10‐year‐old males versus females, effect size (Hedges' g) = 0.679. *These track meets were not held in 2020 due to the COVID‐19 pandemic.

400 m: Finishing times in the 400m race for the 8 and under males (72.94 ± 6.53 s) were faster (*p* = 0.000; Hedges' g = 0.640) than for the 8 and under females (76.78 ± 5.44 s). The range of finishing times in the 400‐m race for the 8 and under males was 62.48–97.17 s (Figure 2, upper panel), while the range for the 8 and under females was 67.33–88.72 s. Fifteen males in the 8 and under age group had faster 400m finishing times than the fastest female in the 8 and under age group.

Finishing times in the 400‐ m race for the 9–10‐year‐old males (64.76 ± 2.44 s) were faster (*p* = 0.000; Hedges' g = 0.664) than for the 9–10‐year‐old females (67.37 ± 4.35 s). The range of finishing times in the 400‐m race for the 9–10‐year‐old males was 58.89–80.23 s (Figure 2, upper panel), while the range for the 9–10‐year‐old females was 58.97–82.72 s. One male in the 9–10‐year‐old age group had a faster 400m finishing time than the fastest female in the 9–10‐year‐old age group.

800 m: Finishing times in the 800‐m race for the 8 and under males (168.99 ± 12.00 s) were faster (*p* = 0.000; Hedges' g = 0.834) than for the 8 and under females (180.37 ± 15.22 s). The range of finishing times in the 800‐m race for the 8 and under males was 148.26–202.39 s (Figure 2, lower panel), while the range for the 8 and under females was 154.22–233.37 s. Eight males in the 8 and under age group had faster 800m finishing times than the fastest female in the 8 and under age group.

Finishing times in the 800‐m race for the 9–10‐year‐old males (153.15 ± 8.73 s) were faster (*p* = 0.000; Hedges' g = 0.679) than for the 9–10‐year‐old females (159.30 ± 9.37 s). The range of finishing times in the 800‐m race for the 9–10‐year‐old males was 138.51–181.77 s (Figure 2, lower panel), while the range for the 9–10‐year‐old females was 145.07–187.47 s. Nineteen males in the 9–10‐year‐old age group had a faster 800m finishing time than the fastest female in 9–10‐year‐old age group.

**FIGURE 3:**
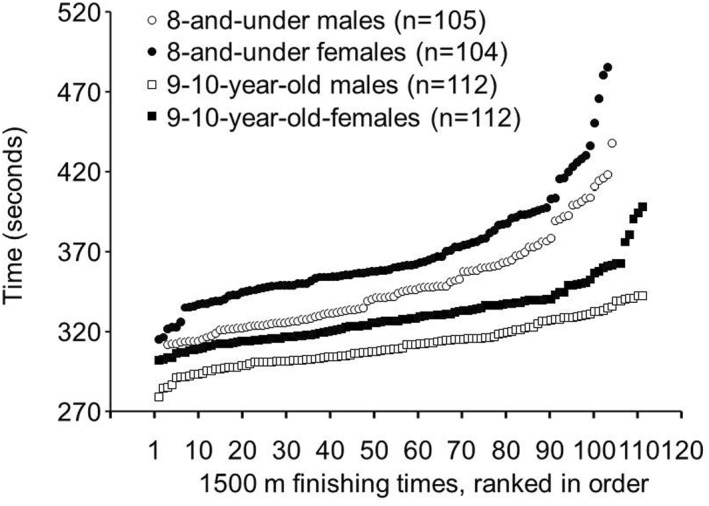
Finishing times from fastest to slowest for the finalists in the 1500‐m race in the 8 and under and 9–10‐year‐old age groups from the USA Track and Field National Youth Outdoor Championships and the USA Track and Field National Junior Olympic Championships during the years 2016–2023*. *p* = 0.000 for 8 and under males versus females, effect size (Hedges' g) = 0.676. *p* = 0.000 for 9–10‐year‐old males versus females, effect size (Hedges' g) = 1.089. *These track meets were not held in 2020 due to the COVID‐19 pandemic.

1500 m: Finishing times in the 1500‐m race for the 8 and under males (347.10 ± 29.24 s) were faster (*p* = 0.000; Hedges' g = 0.676) than for the 8 and under females (368.24 ± 33.23 s). The range of finishing times in the 1500‐m race for the 8 and under males was 301.95–437.73 s (Figure 3), while the range for the 8 and under females was 314.72 485.30 s. Ten males in the 8 and under age group had faster 1500m finishing times than the fastest female in the 8 and under age group.

Finishing times in the 1500‐m race for the 9–10‐year‐old males (311.58 ± 14.40 s) were faster (*p* = 0.000; Hedges' g = 1.089) than for the 9–10‐year‐old females (330.11 ± 19.27 s). The range of finishing times in the 1500‐m race for the 9–10‐year‐old males was 278.63–342.37 s (Figure 3), while the range for the 9–10‐year‐old females was 301.94–398.11 s. Thirty‐four males in the 9–10‐year‐old age group had a faster 1500m finishing time than the fastest female in the 9–10‐year‐old age group. Finishing times in the 1500‐m race for the 9–10‐year‐olds were available for 56 males from the USATF Junior Olympics and 56 males from the USATF National Championships.

## 4 DISCUSSION

In this evaluation of finishing times for finalists in the 100, 200, 400, 800, and 1500m events in the USATF National Youth Outdoor Championships and Junior Olympic National Championships from 2016 to 2023, males in the 8 and under and 9–10‐year‐old age groups had faster average finishing times than females, and the fastest times for males were faster than the fastest times for females. These sex‐based differences in running performance corresponded with moderate to large effect sizes indicate that the differences are of considerable practical importance. These findings therefore indicate that there are consistent and meaningful sex‐based differences in track running performance in the 100, 200, 400, 800, and 1500m events before puberty.

The faster prepubertal running times for males in the present data are comparable to what has been reported previously. Handelsman (2017) used an online source of world record race times for distances of 50, 60, 100, 200, 300, 400, 500, 600, 800, 1000, 1500m, 1 mile, 2000m, 3000m, and 2 miles and reported that the prepubertal sex‐based differences for all events combined were 3.0%. The School Sport Australia Track & Field Championships records for all‐time best performances (as of December 2016) (School Sport Australia) indicate that for 10‐year‐olds in the 100, 200, and 800m races, the males run faster than females in every event by 3.2 ± 1.7%. The USATF Youth records for all‐time best performances (as of December 19, 2018) (USATF, 2018) indicate that males run faster in every event for the 100, 200, 400, 800, and 1500m events with males in the 8 and under age group running 3.7 ± 2.4% faster than females, and in the 9–10‐year‐old age group the males run 3.5 ± 3.0% faster than females. The USATF Junior Olympics records for all‐time best performances (as of March 27, 2019) (USATF, 2019) indicate that males under age 10 run faster than females in every event for the 100, 200, 400, 800, and 1500m events with males in the 8 and under age group running 4.6 ± 2.5% faster than females, and in the 9–10‐year‐old age group the males run 2.1 ± 0.8% faster than females. The Amateur Athletic Union Junior Olympics records for all‐time best performances (as of November 22, 2023) (AAU) indicate that males under age 10 run faster than females in every event for the 100, 200, 400, 800, and 1500m, with 8 and under males running 3.0 ± 1.3% faster, 9‐year‐old males running 3.7 ± 0.7% faster, and 10‐year‐old males running 4.1 ± 2.1% faster than females of the same ages. In 2018, Millard–Stafford (Millard‐Stafford et al., 2018) reported that in adults the all‐time best records from running distances of 100m to marathon for males are 9.1%–13.9% faster than females, while more recently Hunter et al. (2023) indicates that adult males run 9.5%–12.3% faster than females. The race time differences between males and females in children under age 10 are smaller than the differences observed in adults, primarily due to the testosterone driven changes that occur during male puberty and the estrogen driven changes that occur during female puberty (Bassett et al., 2020; Handelsman, 2017; Handelsman et al., 2018). However, the present and previous data indicate that there are sex‐based differences in running performance before the onset of puberty, with males consistently running faster than females.

It has been claimed in scholarly sources (Ervin et al., 2013; Handelsman, 2017; Handelsman et al., 2018; Safer, 2022), legal documents (A.M. v. Indianapolis Public Schools, 2022; Roe v. UHSAA, 2022), and in popular media (Mertens, 2022) that there are no differences in athletic performance prior to puberty. However, the normative data from the Presidential Fitness test (President’s Challenge Qualifying standards) and numerous investigations indicate that males as young as 6 years old perform better than similarly aged girls on physical fitness tests for muscle strength, muscle endurance, aerobic fitness, and running speed (Catley & Tomkinson, 2013; Eiberg et al., 2005; Fuhner et al., 2021; Golle et al., 2015; Tambalis et al., 2016; Tomkinson et al., 2017, 2018; Vanhelst et al., 2020). For example, Tomkinson (Tomkinson et al., 2018) indicates that at ages 9 and 10 males run ∼3.0% faster than females during the final stage of a 20‐m shuttle run. Golle (Golle et al., 2015) observed that 9‐year‐old males were ∼3.0% faster than females when completing a 50‐m sprint test and ran ∼10% further during a 9‐min running test. While physical fitness testing does not necessarily simulate athletic competition, and physical fitness testing does not always predict athletic performance, it is unquestionable that higher physical fitness predisposes an athlete to better performance in competition. The present data showing faster competitive running performance in males compared to same aged females under age 10 is consistent with the findings of male advantages in physical fitness that support running performance (i.e., muscle strength, muscle endurance, aerobic fitness, and running speed).

In contrast to the consistent prepubertal sex‐based differences in running performance in the present data and all‐time best records (AAU; School Sport Australia; USATF, 2018; USATF, 2019), the differences in swimming performance are less clear. The sex‐based differences in the 10 and under records for all‐time best performances from the USA Swimming (as of November 22, 2023), indicating that males are faster than females in 20 out of 23 events with an average sex‐based difference of ∼1.8% (USA Swimming). However, Senefeld et al. (2019) reported that prior to age 10, the top 5 female swimmers were faster than comparable males, and there was no performance difference between the 10th–50th ranked males and females. In comparison to running, the smaller to nonexistent prepubertal sex‐based differences in swimming may be partially explained by noting that swimming is a learned skill that requires access to facilities and coaching to a much greater extent than does the innate skill of running (Olaisen et al., 2018). Furthermore, Duke et al. (2023) observed that in addition to sex, school type was a major factor influencing swimming skills since some schools, particularly smaller or less affluent schools, lack aquatic facilities. These authors also observed that frequency of participation in aquatic activity and prior negative aquatic experiences also contributed to the smaller prepubertal sex‐based differences in swimming performance than other sports. It also appears that more females participate in competitive swimming as children than do males (Senefeld et al., 2019), which can increase the talent pool and level of competition resulting in better performance. It is also speculated that females are more likely to focus solely on swimming and are more likely to enjoy training for competitive swimming (Senefeld et al., 2019), which may also result in better performance. Therefore, it appears plausible that increasing the time and type of swimming practice may allow females to overcome some, but not all, of the prepubertal sex‐based anatomical and physiological differences causing sex‐based differences in athletic performance. However, there is evidence indicating that when male and female children engage in the same amount and intensity of physical activity, the males develop greater physical fitness (Dencker et al., 2007; Eiberg et al., 2005), which may predispose males to better athletic performance.

It has been suggested that the prepubertal sex‐based differences in physical fitness and athletic performance are due to males participating in a greater amount of physical activity, higher intensity physical activity, and more sports than do females (Hunter et al., 2023). Supporting this suggestion, Chen et al. (Chen et al., 2018) observed in fifth‐grade children that males spend more time engaged in unstructured physical activity than females, but there was not a sex‐based difference in time spent in organized physical education classes, sports, and dance. Hyde et al. (2020) reported that 60.9% of male children and 54.4% of female children engaged in organized sports. Belcher et al. (2010) reported that in children aged 6–11‐years‐old, males participate in more moderate to vigorous physical activity than do females. Collectively, these sources support the notion that prepubertal sex‐based differences in athletic performance can at least be partially explained by higher levels of sport participation and more strenuous physical activity in males than in females. However, in 6–7‐year‐old children (Eiberg et al., 2005) and in 8–11‐year‐old children (Dencker et al., 2007), the sex‐based differences in maximal oxygen consumption and body composition were not entirely explained by differences in physical activity. While greater engagement in physical activity and sports is likely to contribute to better sports performance (providing that overtraining syndrome is avoided), and there are some data that indicate in children males engage in more sports and physical activity than females, it is not possible to disregard prepubertal sex‐based differences in anatomy and physiology also as causative factors for prepubertal sex‐based differences in athletic performance.

McManus and Armstrong (McManus & Armstrong, 2011) presented a very thorough review of the physiological differences between young male and female athletes, including before puberty, and how these differences facilitate male advantages in athletic performance. For example, McManus and Armstrong (McManus & Armstrong, 2011) report that males have ∼10% more lean body mass than females before puberty, which is also supported by a review from Staiano and Katzmarzyk (Staiano & Katzmarzyk, 2012). It is well known that having more lean body mass relative to fat mass facilitates better athletic performance (Hunter et al., 2023; Joyner & Coyle, 2008). McManus and Armstrong (McManus & Armstrong, 2011) also report that before puberty, males have larger and more efficient hearts and lungs than females, which can facilitate better athletic performance. There are also differences in the width of the ischium and acetabular regions of the pelvis between males and females before puberty (Leong, 2006), which may contribute to male advantages in running mechanics and efficiency similar to what has been observed in adults (Daniels & Daniels, 1992; Ferber, Davis, & Williams, 2003). Before puberty, there are no differences in circulating testosterone or hemoglobin concentrations between males and females (McManus & Armstrong, 2011; Senefeld et al., 2020). However, there are sex‐based differences in lean body mass, cardiac size and function, lung size and function, and pelvic dimensions, all of which may contribute to the faster running times demonstrated by males with ages 10 and under compared to females of the same age in the present and previous data.

As the present data are based on track meet records, these data offer no insight into the descriptive characteristics of the athletes other than sex and age. The event records also must be taken at face value, with no knowledge of the accuracy and precision of the timing used for each event. However, the track meets all occurred under the auspices of USA Track & Field, which has standards for timing and for athlete eligibility. Although these track meets were labeled as national championships, it is not clear that the best juvenile athletes from across the country are represented among the participants (which is a common challenge in children's sports due to travel costs and family circumstances associated with attending an event outside the immediate local area). However, these limitations can also be applied to the data evaluated by Handelsman (2017) and are inherent to the USA Track and Field Youth records (USATF, 2018; USATF, 2019), the AAU Track and Field youth records (AAU), the School Sport Australia records (School Sport Australia), and indeed to any comparison of youth sports records.

## 5 CONCLUSION

In conclusion, although some have stated that sex‐based differences in athletic performance do not arise until puberty, the present data indicate that in the 8 and under and 9–10‐year‐old age groups males run faster than females in distances of 100, 200, 400, 800, and 1500m. While some females in these age groups are faster than some males, the average male finalists are faster than the average female finalists, and the fastest males are faster than the fastest females. As running is a key component of many sports, these sex‐based differences between prepubertal males and females should be considered when sport governing bodies and policy makers consider the issue of sex‐based sporting categories.

## CONFLICT OF INTEREST STATEMENT

Dr. Brandon Shaw and Dr. Ina Shaw have no conflicts of interest to declare. Dr. Greg Brown declares that he is currently serving as an expert witness in six different legal cases in the United States regarding the inclusion of transgender identified males (i.e., trans women) in female sports. No funding that supported this project as part of service as an expert witness was received, his declarations as an expert witness do not include the data presented in this manuscript, and his service as an expert witness is not reliant upon publishing this manuscript.
